# Effects of In Vitro Digestion on the Content and Biological Activity of Polyphenols from *Acacia mearnsii* Bark

**DOI:** 10.3390/molecules23071804

**Published:** 2018-07-20

**Authors:** Xiao Chen, Jia Xiong, Lingxiao He, Yu Zhang, Xun Li, Liping Zhang, Fei Wang

**Affiliations:** 1 Jiangsu Key Laboratory for the Chemistry and Utilization of Agro-Forest Biomass, College of Chemical Engineering, Nanjing Forestry University, Nanjing 210037, China; 13770765711@163.com (X.C.); a6410@126.com (L.H.); yuzhang@njfu.edu.cn (Y.Z.); xunlee@163.com (X.L.); 2 Plants for Human Health Institute, Food Bioprocessing and Nutrition Sciences Department, North Carolina State University, North Carolina Research Campus, Kannapolis, NC 28081, USA; jxiong5@ncsu.edu; 3 College of Materials Sciences and Technology, Beijing Forestry University, Beijing 100083, China; zhanglp418@163.com

**Keywords:** *Acacia mearnsii*, proanthocyanidins, in vitro digestion, antioxidant, antidiabetic, HPLC/MS

## Abstract

The stability and bioaccessibility of polyphenol from *Acacia mearnsii* bark were measured at various stages during in vitro simulated digestion. Subsequently, the changes in the total polyphenol content (TPC) and biological activity were studied. The results showed that the phenolic compounds from *A. mearnsii* remained stable, and TPC underwent few changes during gastric digestion. Nonetheless, intestinal digestion led to the degradation of proanthocyanidins (PAs) and a significant decrease in TPC (26%). Degradation was determined by normal-phase HPLC and gel permeation chromatography. Only monomers, dimers, and trimers of flavan-3-ols were identified in the serum-accessible fraction for characterization of their bioaccessibility. The results also indicated the obvious antioxidant capacity of PAs from *A. mearnsii* bark, and ~53% of the α-glucosidase–inhibitory effect was preserved. All these findings show that PAs from *A. mearnsii* bark as a native plant source may be particularly beneficial for human health as a natural nutritional supplement.

## 1. Introduction

Proanthocyanidins (PAs), also known as condensed tannins, are oligomers or polymers of flavan-3-ols linked through interflavan bonds and are commonly found in fruits, nuts, cereals, seeds, wine, bark, and chocolate [1,2]. PAs are well-known for their antioxidant properties and other physiological actions that reduce risk factors of certain types of diseases [3]. Numerous studies have revealed a relationship between the consumption of fruit, vegetables, or cocoa and beneficial effects on the cardiovascular disease risk; these effects are reportedly due to the presence of various antioxidants, particularly polyphenols [4,5,6].

*Acacia mearnsii* bark extract, an important renewable industrial raw material, has traditionally been used in leather tanning and adhesive processing [7,8] and is now recognized to have strong physiological effects, such as antioxidant, anti-inflammatory, and enzyme-inhibitory activities [9,10,11]. In our previous study, we observed that *A. mearnsii* bark extract not only has a strong antioxidant activity and certain antitumor properties but also shows good bioavailability in vitro. In addition, *A. mearnsii* bark extract has an antidiabetic effect in vitro [10]. Based on their preferable biological activities and a considerable potential to serve as a food-grade bioactive component (“nutraceutical”) in functional food products, PAs from *A. mearnsii* bark should be investigated regarding potential practical applications.

One of the principal topics concerning the beneficial effects of these PAs is their bioavailability and metabolic fate. The bioavailability of a dietary compound depends upon its digestive stability and the efficiency of its transepithelial passage [12,13]. Determining the true bioavailability of any class of phytochemicals requires collection of data concerning the absorption, metabolism, tissue and organ distribution, and excretion of these compounds. Performing such studies on animals or human subjects is complicated and expensive and also raises moral and ethical questions [14]. Therefore, in vitro methods are useful to study the stability of compounds under simulated gastrointestinal conditions. Various studies have indicated that bioavailability differs greatly from one polyphenol to another and depends on a dietary source for some compounds [13]. For instance, PAs from cocoa may be degraded into monomeric and dimeric forms during their transit in the stomach with simulated gastric juice (pH 2–4) [15], but PAs from *Choerospondias axillaris* peels are stable, and most ingested PAs should reach the small intestine and be accessible for absorption or metabolism [13]. Various studies show the effect of in vitro gastrointestinal digestion on the stability of phenolic compounds from beverages [16], solid food matrices [17], and wine [18] as well as pure phenolic compounds [19]. Nevertheless, very few studies have been carried out on PAs from bark although they have been found to have strong biological activity [10,11].

When vegetables, fruits, and their derivatives are ingested directly, polyphenols appear in the gastrointestinal tract at a high concentration. Moreover, polyphenols not only act as antioxidants to protect the gastrointestinal tract from oxidative damage but also inhibit the activity of α-glucosidase, the key enzyme that metabolizes nonabsorbable oligosaccharides into monosaccharides in the small intestine. Inhibiting this enzyme can control blood glucose levels in type 2 diabetes after consumption of foods with a high glycemic index [20].

The aim of this study was to determine the effects of gastrointestinal digestion on stability, bioaccessibility, and α-glucosidase–inhibitory activity of PAs from *A. mearnsii* bark. Simultaneously, the profiles of the bioaccessible PAs were investigated by UFLC-ESI-QTOF-MS^2^.

## 2. Results and Discussion

### 2.1. Changes in the Total Polyphenol Content (TPC) and Stability of PAs

**Figure 1:**
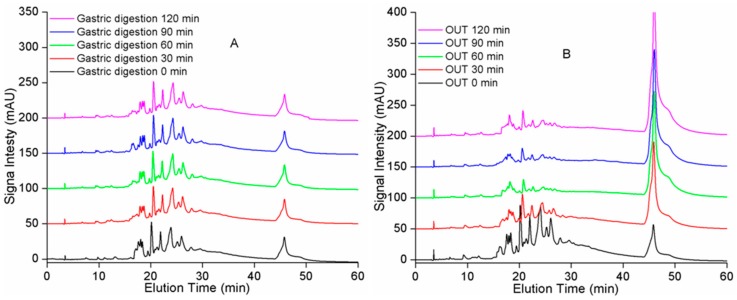
Representative normal-phase HPLC/VWD chromatograms of samples from *A. mearnsii* bark at different time points during the simulated gastric-intestinal digestion, (**A**) gastric digestion; (**B**) intestinal digestion. The OUT samples represent the solution outside the tubing.

The TPC of the bioaccessible extract in both simulated gastric and intestinal digesta is shown in Table 1, and the data of control without enzymes are displayed in Table S1. The TPC of the nondigested sample was 6.08 mg/mL. After simulated gastric digestion, TPC decreased by 10% from 6.08 to 5.45 mg/mL. The TPC of samples did not change significantly between minutes 90 and 120 but showed a small reduction between minutes 0 and 60 and a slight increase between minutes 60 and 90, while the data of control without enzymes showed very little decrease. This may be because protein–polyphenol interactions are typically relatively weak and reversible [21,22,23]. Given that larger PAs separate according to their degree of polymerization (DP) in a silica column, normal-phase HPLC (NP-HPLC) was conducted to characterize the DP as presented in Figure 1. The NP-HPLC chromatogram showed no change upon gastric digestion (Figure 1A). TPC underwent a slight decrease. The above results pointed to the high stability of PAs, and that most of the ingested PAs should reach the small intestine and be accessible for absorption or metabolism. Other studies have also revealed that gastric digestion has no substantial effect on polyphenols and PAs [13,24], but some studies have shown that the PA oligomers in cocoa liquor are completely hydrolyzed during gastric digestion, leading to an increase in the amounts of monomers, and mainly dimers [25]. Therefore, these data suggest that the stability of PAs during gastric digestion depends on the dietary source.

After simulated digestion by the stomach, the extract was subjected to intestinal conditions for 2 h by means of pancreatic enzymes and bile salts. During this phase, TPC of the OUT sample (compounds that did not cross a dialysis barrier) immediately sharply decreased (Table 1). Then, TPC decreased between minutes 0 and 60 and increased between minutes 60 and 90, resembling the results of the gastric-digestion stage, while the TPC of control also showed little decrease between 0 and 120 min (Table S1). Nevertheless, there was a ~26% loss compared to the nondigested sample. Meanwhile, NP-HPLC chromatograms (Figure 1B) also indicated a large change because the signal intensity manifested marked weakening at 20–30 min, and the hump signal increased at about 47 min. Most researchers have inferred that the loss of TPC during simulated intestinal digestion was mainly due to the chemical conditions (mildly alkaline) because polyphenols are highly sensitive to degradation under alkaline conditions owing to the conversion of phenolics into other unknown or undetected compounds [24]. In this study, we found a sharp decrease at the initial stage of intestinal digestion when pH was 5.5 (weakly acidic). Therefore, the phenolic compounds should have been stable. Our earlier studies have also revealed that α-amylase and α-glucosidase are strongly inhibited by samples from *A. mearnsii* bark. Meanwhile, because protein–polyphenol interactions are typically relatively weak and reversible [23], TPC decreased between minutes 0 and 60 and increased between minutes 60 and 90.

### 2.2. Gel Permeation Chromatography (GPC) Analysis

**Figure 2:**
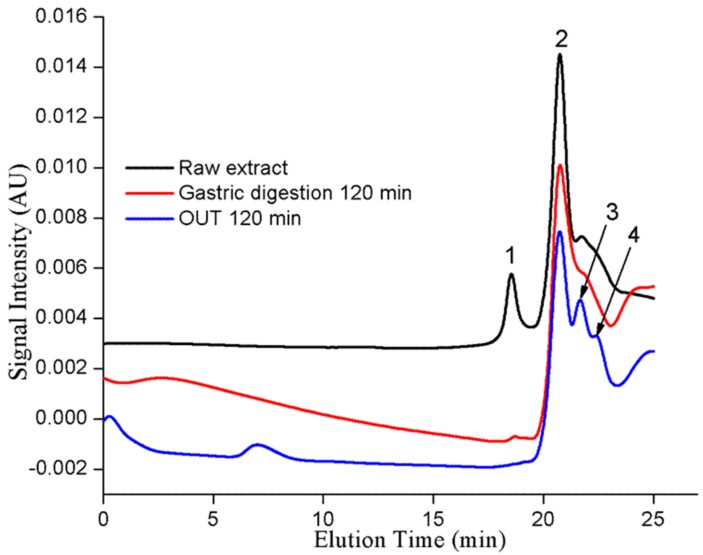
Representative gel permeation chromatograms of the raw extract, gastric digestion at 120 min, and OUT 120 min samples prepared in tetrahydrofuran.

GPC provided molecular weight distribution profiles of the raw extract, postgastric, and OUT 120 min samples as shown in Figure 2. The chromatogram of postgastric digestion uncovered a slight change as peak 1 at 18.5 min turned into a very small peak compared to that of the raw extract, but peak 2 did not change, probably because the PAs with a larger molecular size bind to enzymes more easily. Other researchers have also found a slight decrease in mDP after gastric digestion [13]. Nonetheless, the chromatogram of OUT 120 min samples revealed a large change. Compared to peaks in the raw extract, peak 1 disappeared and peaks 3 and 4 appeared. Peaks 3 and 4 may represent newly generated degradation products of PAs. Of note, the negative signal value of the GPC chromatogram may be due to the presence of macromolecular pepsin and pancreatin.

### 2.3. Changes in PA Bioaccessibility during Digestion

During the intestinal stage, the TPC of IN samples (compounds that did cross a dialysis barrier) increased with incubation time from 0.19 to 1.09 mg/mL between minutes 30 and 120, but it was far below that of the OUT samples and nondigested extract. The bioaccessibility of PAs from *A. mearnsii* bark was approximately 17.9%. Other researchers have established different accessibility rates, such as 13.7% accessibility rate of PAs from *C. axillaris* peels, and less than 10% for (+)-epicatechin; this variation was influenced by molecular weight and PA stability. Interactions between phenolic compounds and other constituents may favor the formation of complexes with low solubility or large molecular weight that cannot cross the dialysis membrane [26]. It has been reported that PAs are absorbed through passive diffusion without any transporters and are unlikely to pass through the lipid bilayer via the transcellular pathway due to the large number of hydrophilic hydroxyl groups [27]. Therefore, the above reasons explained the low bioaccessibility previously observed in vivo and in vitro [26,27].

We found only oligomeric PAs in the IN samples by NP-HPLC (not shown), and then identified them on a Shimadzu UFLC (Shimadzu Corp., Kyoto, Japan) system connected with a Triple TOF 4600 (AB Sciex, Framingham, MA, USA). All chromatography peaks were identified by mass spectra (Table 2) and were compared with the results of previous studies [2]. The product ions of *m*/*z* 289 and 305 (Figures S1 and S2) were tentatively identified as catechin and gallocatechin by comparing with the MS of their authentic samples in other studies [2]. The product of the *m*/*z* 561 ion that corresponded unambiguously to a fisetinidol–catechin conjugate yielded the catechin *m*/*z* 289 molecular fragment as a base peak, and the diagnostic fisetinidol extender unit fragment at 161 was found (Figure S3), as would be expected from fission of a fisetinidol–catechin interflavanyl bond. We also found the retro-Diels–Alder fission (RDA) fragment of the catechin starter unit with the fisetinidol extender unit intact at *m*/*z* 409. The *m*/*z* 577 molecular fragment could either represent a fisetinidol–gallocatechin or robinetinidol–catechin (Figure S4). Because robinetinidol (*m*/*z* 287) and catechin (*m*/*z* 289) extender units were observed in the fragmentation pattern, and the diagnostic fisetinidol and robinetinidol extender unit fragments at *m*/*z* 161 and 177 were detected, we assumed the presence of fisetinidol–gallocatechin and robinetinidol–catechin. The product ion scan of the *m*/*z* 593 peak, which corresponded unambiguously to robinetinidol–gallocatechin, gave gallocatechin (*m*/*z* 305) as the base peak (Figure S5). We also observed RDA fragmentation of the gallocatechin starter unit at *m*/*z* 425 with the robinetinidol extender unit intact and an RDA-H_2_O molecular fragment at *m*/*z* 407, and the *m*/*z* 177 ion represents a robinetinidol extender unit. The *m*/*z* 833 peak in the mass spectrum corresponded unambiguously to a fisetinidol–catechin–fisetinidol trimer (Figure S6). Fragmentation gave the expected diagnostic catechin (*m*/*z* 289), fisetinidol (*m*/*z* 161), and [M–152] RDA (*m*/*z* 681) molecular fragments. The *m*/*z* 561 molecular fragment (fisetinidol–catechin) corresponded to the loss of one fisetinidol moiety. The product *m*/*z* 849 trimer peak corresponded to fisetinidol–catechin–robinetinidol or fisetinidol–gallocatechin–fisetinidol (Figure S7). RDA fragments at *m*/*z* 697 in the tandem mass spectrometry (MS^2^) analysis of the *m*/*z* 849 trimer, corresponding to the loss of 152 Da, indicated the presence of catechin starter units. Gallocatechin (*m*/*z* 305) and the diagnostic robinetinidol extender unit fragments at *m*/*z* 177 pointed to the presence of fisetinidol–gallocatechin–fisetinidol. The *m*/*z* 865 trimer peak corresponded to fisetinidol–gallocatechin–robinetinidol or to robinetinidol–catechin–robinetinidol (Figure S8). The RDA fragments at *m*/*z* 713 indicated the loss of 152 Da in the MS^2^ analysis of the *m*/*z* 865 trimer, and the robinetinidol–catechin (*m*/*z* 577) MS^2^ fragments indicated that the *m*/*z* 865 trimer consists of robinetinidol, catechin, and robinetinidol. The *m*/*z* 881 peak, which corresponded unambiguously to a robinetinidol–gallocatechin–robinetinidol trimer, gave the expected diagnostic gallocatechin (*m*/*z* 305), robinetinidol (*m*/*z* 177), and [M − 168] RDA (*m*/*z* 713) molecular fragments. The *m*/*z* 593 molecular fragment (robinetinidol–gallocatechin) was suggestive of the loss of one robinetinidol moiety (Figure S9). The results showed that there were only “B-type” PAs as monomers, dimers, and trimers in the serum-accessible fraction (IN sample). This result indicated that the DP had a large effect on bioactivity; this finding is consistent with the results from in vitro and in vivo models, namely, that PAs with a DP over 4 are not absorbable across the gut barrier because of their large molecular dimensions [28]. Most ingested PAs reach the colon, being either extensively depolymerized or metabolized by the gut microbiota, and PAs have been found to optimize the diversity and dominance patterns of intestinal microflora in mice [29,30].

### 2.4. Changes in Antioxidant Activities of Digesta

Changes in the antioxidant activity were measured by means of 2,2-diphenyl-1-(2,4,6-trinitrophenyl)hydrazyl (DPPH) and 2,2′-azinobis-(3-ethylbenzthiazoline-6-sulphonate) (ABTS) radical-scavenging activities during the in vitro gastrointestinal digestion. Results are given in Table 3. The gastric digestion with pepsin-HCl (pH 2.0) was found to have no substantial effect on the antioxidant activity measured by DPPH and ABTS assays with a 12% and 10% decrease, respectively, as compared to a nondigested sample, which manifested the highest stability of PAs from *A. mearnsii* bark. A significant loss of antioxidant activities occurred after gastrointestinal digestion: reductions of 42% and 30%, respectively, because of the partial degradation of phenolic compounds. As for the IN samples, antioxidant capacity increased with digestion time as more monomers, dimers, and trimers slowly permeated into the cellulose dialysis tube, but the antioxidant capacity was much lower than that of OUT samples and gastric digesta. This is mainly because phenolic compounds with a large molecular weight or phenolic compounds bound to other constituents result in the formation of large complexes that cannot pass through the small pores in the dialysis membrane [13]. The above data also indicated that phenolic compounds were the major contributors to antioxidant properties of the *A. mearnsii* extract, as demonstrated in other studies [31].

The results were expressed as nanoliters of digested extract achieving half of the clearance rate. Values followed by the same letter in the same column are significantly different from each other (*p* < 0.05). Data are means of three independent analyses ± SD.

### 2.5. Changes in α-Glucosidase–Inhibitory Activities of Digesta

**Figure 3:**
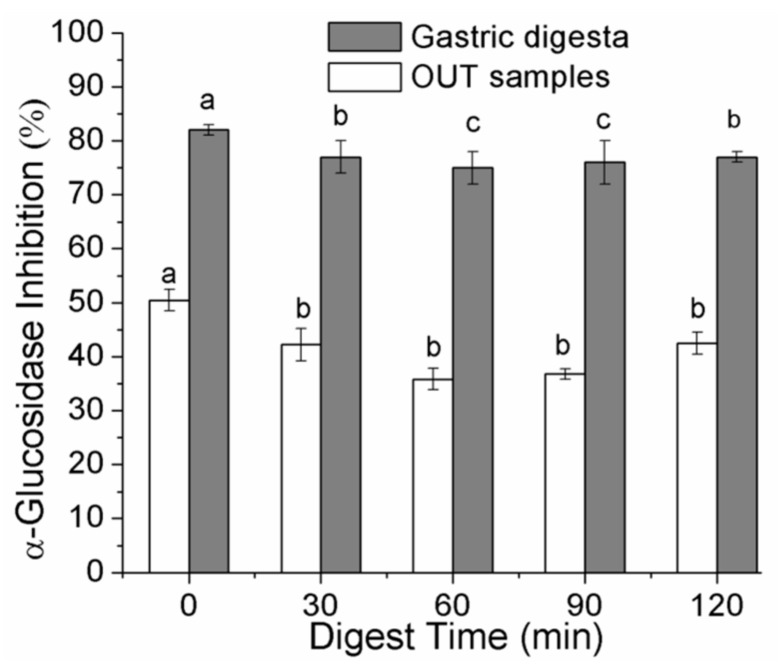
Percent α-glucosidase inhibition for gastric digesta and OUT samples at different time points during in vitro gastrointestinal digestion. The mean values reported at each digestion stage-those with a common superscript (a–c) are not significantly different from each other (*p* < 0.05). Data are means of three independent determinations ± SD.

Because bioavailability is not necessary for PAs of any size to exert activity by inhibiting digestion of lipids or carbohydrates in the gastrointestinal lumen [32], we measured the α-glucosidase–inhibitory effect of gastric digesta and OUT samples. All the samples were diluted 100-fold with phosphate buffer (0.1 M, pH 6.9). Results are illustrated in Figure 3. After simulated gastric digestion, the inhibitory capacity of the *A. mearnsii* bark extract remained constant and showed ~77% of the activity, and only decreased by 2.9% compared with the control; this finding was consistent with the high stability of PAs from *A. mearnsii* bark. Then, the extract was subjected to intestinal digestion, and the activity was ~42% of that of the control and thus decreased by approximately 47%. These data confirmed that the inhibitory capacity diminished after partial degradation of PAs. The results indicated that the gastric digesta remained stable and exerted obvious inhibition of α-glucosidase at a low concentration. Consequently, *A. mearnsii* bark from native plant sources is particularly promising as a natural alternative to antidiabetic drugs, representing an attractive tactic for controlling hyperglycemia and the development of functional foods for patients with diabetes.

## 3. Materials and Methods

### 3.1. Materials and Reagents

*A. mearnsii* bark was kindly supplied by Crown Forest Farm in Guangxi Province, China. α-Glucosidase, *p*-nitro-phenyl-α-d-glucopyranoside, pepsin from porcine gastric mucosa, DPPH, ABTS, and cellulose dialysis membrane (molecular weight cutoff of 12 kDa) were acquired from Sigma-Aldrich (St. Louis, MO, USA). Pancreatin (USP) from porcine pancreas was purchased from Aladdin (Shanghai, China), and bile salts from Solarbio Life Science Company, Ltd. (Beijing, China). A C-18 solid-phase extraction (SPE) column (2 g capacity) was purchased from Navigator Lab. Instrument Company Ltd. (Tianjin, China), whereas HPLC-grade acetic acid, methanol, dichloromethane, acetonitrile, trifluoroacetic acid, and tetrahydrofuran from Tedia (Fairfield, OH, USA). All other reagents and solvents used were analytical grade.

### 3.2. Extraction of PAs

The dried raw *A. mearnsii* bark was ground up with a small industrial pulverizer. The powder was passed through a No. 5 mesh with sieve opening of 4 mm. Of the obtained powder, 500 g was resuspended in 50% (*v*/*v*) ethanol (5 L) and stirred at 300 rpm and 25 °C for 12 h. The extraction was performed twice. Then, the combined extracts were evaporated to remove the organic solvent. The aqueous phase was recovered and washed with hexane and dichloromethane three times each to remove nonpolar material, and then the organic solvents were evaporated under vacuum. Finally, the aqueous phase was lyophilized to dryness to obtain the extract. These resulting extracts were stored at −20 °C until further analysis.

### 3.3. Simulated In Vitro Gastric and Intestinal Digestion

The reported protocol of in vitro bioavailability assays consists of two sequential steps: initial pepsin + HCl digestion for 2 h at 37 °C to simulate gastric conditions, followed by digestion with bile salts and pancreatin for 2 h at 37 °C to simulate the conditions in the small intestine [13]. This method has been used to simulate human digestion and absorption of dietary iron from complex meals and shows a significant correlation between the in vitro and in vivo measurements of iron bioavailability [33]. Briefly, for pepsin + HCl digestion, 3 g of the raw extract was dissolved in 300 mL of deionized water, and pH was adjusted to 2.0 by adding 6 M HCl, then pepsin was added at 315 U/mL. Next, the samples were incubated in a 37 °C water bath for 2 h with shaking at 60 rpm. Every 30 min, aliquots (5 mL) of the postgastric digestion were removed for determination of TPC, HPLC, antioxidant activity, and α-glucosidase inhibition. The remaining samples were placed in a 500 mL glass beaker, and 1 M NaHCO_3_ was added to adjust the pH to 5.5. After that, 5 mL of freshly prepared 4 mg/mL pancreatin and 25 mg/mL bile salts were added, segments of cellulose dialysis tubing (molecular weight cutoff of 12 kDa) containing 1 M NaHCO_3_ equivalent were required to titrate the combined pepsin-digest pancreatin-bile extract mixture to pH 7.5 in the glass flask. Every 30 min, aliquots (5 mL) of the solution outside and inside the dialysis tubing (referred to as “OUT samples” and “IN samples”, respectively) were taken to represent the material that remained within the gastrointestinal tract and the fraction that was accessible for absorption, respectively.

All the samples were heated at 90 °C for 1 min to terminate the digestion process. Controls without added enzymes were run in parallel to differentiate between the effects due to the presence of enzymes from those that may be caused by the chemical environment in the assays.

### 3.4. Analytical Assays

#### 3.4.1. Clean-Up of the IN and OUT Samples

A C18 solid-phase extraction (SPE) column was employed to remove the bile salts in the IN and OUT samples. The clean-up procedure has been described previously [13]. Briefly, the samples were acidified to 0.5% (*v*/*v*) by addition of a 10% trifluoroacetic acid (TFA) solution, then centrifuged at 9694 *g* for 8 min; the clear liquids were applied to a C18 SPE column that had been pre-equilibrated with 0.5% (*v*/*v*) aqueous TFA. First, the column was washed with three volumes of 0.5% aqueous TFA, then the bound material was eluted with 0.5% (*v*/*v*) TFA in water and acetone (50:50, *v*/*v*). All the chromatographic fractions were evaporated to remove the organic solvent at 45 °C, and the aqueous phase was lyophilized to dryness. The freeze-dried samples were reconstituted with 5 mL of methanol for subsequent TPC, HPLC, antioxidant activity, and α-glucosidase inhibition analyses.

#### 3.4.2. Determination of TPC and NP-HPLC/VWD

TPC was determined by the Folin–Ciocalteu assay adapted to the 96-well microplate format [34]. Briefly, 50 μL of a diluted sample aqueous solution and standard were added to 150 μL of the freshly prepared Folin–Ciocalteu reagent (1:4 (*v*/*v*) in distilled water) in a 96-well microtiter plate. After incubation for 10 min at 37 °C, 50 μL of a saturated sodium carbonate solution was added into each well, and the plate was incubated for 10 min at 37 °C. Absorbance of each solution was measured at 765 nm on an ELISA reader (Bio-Tek, Washington, DC, USA). The results were expressed as milligrams of gallic acid per milliliter of digested extract using a calibration curve.

According to previously described methods, the ternary mobile phase consisted of (A) dichloromethane, (B) methanol, and (C) acetic acid, and water (1:1, *v*/*v*) [35,36]. The linear gradient was increased as follows: 0–20 min, 14.0–23.6% B; 20–30 min, 23.6–27.4% B; 30–40 min, 27.4–30.0% B; 40–45 min 30–86.0% B; 45–60 min, 86.0% B isocratic. A constant 4.0% concentration of solution C was kept throughout. The samples were filtered through a 0.22 μm membrane before separation on a Luna silica column (250 × 4.6 mm, 100 A; Phenomenex, Torrance, CA, USA) by means of a normal-phase Agilent 1260 HPLC system, with detection at 280 nm and an injection volume of 10 μL. The elution was performed at room temperature at a flow rate of 1 mL/min.

#### 3.4.3. GPC

We subjected 2 mL of postgastric digesta (120 min) and OUT sample (120 min) to vacuum freeze-drying to remove the methanol and dissolved the residue in 2 mL of tetrahydrofuran for GPC analysis. This analysis was performed on a Waters 1515 system, with UV detection at 280 nm. A 300 × 7.8 mm, 10 μm i.d., Styragel HT3 column and a 300 × 7.8 mm, 10 μm i.d., Styragel HT4 column (Waters, Milford, MA, USA) were connected in a series. Separation took place on the columns at 30 °C, by means of an isocratic mobile phase of tetrahydrofuran (1 mL/min) and an injection volume of 10 μL. Polystyrene molecular weight standards ranging from 580 to 19,600 Da were prepared in tetrahydrofuran at ~0.4 mg/mL.

#### 3.4.4. PA Identification: UFLC-QTOF-MS^2^

The IN sample during the simulated digestion was separated and analyzed on a UFLC-ESI-QTOF-MS^2^ system. UFLC-VWD analysis was carried out on a Shimadzu UFLC system connected with Triple TOF 4600. Spectral measurements were conducted at 280 nm. The separation was achieved on a Welch Ultimate XB-C18 column (2.1 mm × 100 mm, 3 μm), with detection at 280 nm. According to a previously described method [13], the mobile phases were composed of 0.25% aqueous acetic acid (eluent A) and 0.25% acetic acid in water and acetonitrile (50:50, *v*/*v*; eluent B) at a flow rate of 0.4 mL/min at 30 °C, using a gradient program as follows: from 10% to 25% B (0–5 min), from 25% to 35% B (5–20 min), from 35% to 40% B (20–25 min), from 40% to 50% B (25–35 min), from 50% to 100% B (35–60 min).

MS analysis was performed on a quadrupole time-of-flight tandem mass spectrometer coupled with an electrospray ionization source in negative ion mode. The nebulizer gas was set to 800 L/h at a temperature of 450 °C. The cone gas was set to a flow rate of 50 L/h, and the source temperature was set to 130 °C. The capillary voltage and the cone voltage were set to 3.0 kV and 35 V, respectively.

#### 3.4.5. Antioxidant-Activity Measurements

Antioxidant activity of the digested samples was measured by DPPH and ABTS methods in a 96-well microplate assay [34]. The DPPH radical–scavenging abilities of all the samples were assessed as follows: 20 μL samples diluted in water to different concentrations were added to 200 μL of DPPH· in 80% ethanol (0.1 mM) in a 96-well microplate. After incubation at 37 °C for 20 min in the dark, the absorbance of each well was determined at 490 nm on an ELISA microtiter plate reader.

The ABTS assay was performed following the reported protocol and was adapted to the 96-well microtiter plate format [34]. ABTS was dissolved in water to a concentration of 7 mM. ABTS radical cations were produced by reacting the ABTS stock solution with 2.45 mM potassium persulfate (final concentration) at a ratio of 1.0:0.5 and by allowing the mixture to stand in the dark at ambient temperature for 12–16 h before use [37]. The absorbance of the ABTS^+^ working solution was read at 734 nm on a UV1700 spectrophotometer, and the solution absorbance was adjusted to 0.700 ± 0.020 by diluting with deionized water. Next, 20 μL diluted samples of the aqueous solution with different concentrations were added to 250 μL of the ABTS^+^ working solution in a 96-well microtiter plate. After incubation at 37 °C for 10 min in the dark, the absorbance of each well was determined at 734 nm using an ELISA reader. The results were expressed as nanoliters of digested extract achieving half of the inhibition.

#### 3.4.6. α-Glucosidase Inhibition

The method employed for determining the α-glucosidase–inhibitory effect was described elsewhere [38]. For the α-glucosidase inhibition assay, the gastric fraction and OUT sample were diluted with 0.1 M sodium phosphate buffer (pH 6.9; 1:100, *v*/*v*). Then, in a 96-well plate, 50 μL of samples or buffer control were added to 100 μL of α-glucosidase (1 U/mL) solution and incubated at 37 °C for 15 min. After that, 50 μL of a 5 mM *p*-nitrophenyl-α-d-glucopyranoside solution was added into each well and incubated for 5 min at 25 °C in the dark. The absorbance was read at 405 nm on an ELISA microtiter plate reader. The α-glucosidase–inhibitory activity was expressed as percentage inhibition:

(1) $$\;\mathrm{Inhibition}\;\left(\%\right)=\;\left[(A_{\mathrm{control}}-A_{\mathrm{sample}})/A_{\mathrm{control}}\right]\times 100\;$$

where *A*_sample_ represents the sample absorbance, and *A*_control_ stands for the control sample absorbance.

### 3.5. Statistical Analysis

Samples were analyzed in triplicate and data were expressed as mean ± standard deviation (SD). Significant differences between the means of parameters were determined by using SPSS 20 statistical software (SPSS, Chicago, IL, USA) (*p* < 0.05). Regression analyses and other statistical analyses were conducted in the OriginPro8 software (OriginLab, Northampton, MA, USA).

## 4. Conclusions

In vitro methods were used to study the stability, bioaccessibility, and biological activity of compounds from *A. mearnsii* bark under simulated gastrointestinal conditions. Simulated gastric digestion caused little change in the TPC and mDP of the extracts or in their antioxidant and α-glucosidase–inhibitory activities. After simulated intestinal digestion, TPC and biological activities decreased significantly compared to those of the nondigested extract. This phenomenon was attributed to the degradation of PAs. Only flavan-3-ols monomers, dimers, and trimers were found in the serum-accessible fraction, which means that the extract possesses relatively low bioaccessibility. The results of this study are suggestive of the good potential of polyphenols from the bark of *A. mearnsii* for human health and that these compounds could be incorporated into functional foods.

## Figures and Tables

**Table 1:** Total polyphenol content (TPC) of samples from *A. mearnsii* bark at different time points during the simulated gastric-intestinal digestion.

Digestion Time (min)	TPC of Digestion Samples (mg/mL)		
	Gastric Digestion	OUT Sample	IN Sample
0	5.97 ± 0.05a	4.69 ± 0.03a	0
30	5.47 ± 0.04b	3.86 ± 0.05b	0.19 ± 0.01a
60	5.24 ± 0.03c	3.88 ± 0.02b	0.40 ± 0.02b
90	5.47 ± 0.06b	3.90 ± 0.03b	0.91 ± 0.02c
120	5.45 ± 0.03b	4.49 ± 0.06c	1.09 ± 0.03d

The IN sample represents the solution that was diffused into the dialysis tubing, the OUT sample represents the solution outside of the tubing. Values followed by the different letters (a–d) in the same column are significantly different from each other (*p* < 0.05). Data represent the means of three independent determinations ± SD.

**Table 2:** Components identified of the IN sample from *A. mearnsii* bark after simulated gastric-intestinal digestion.

Retention Time (min)	Compounds	[M − H]^−^ *m*/*z*	Molecular Fragments	In 30 min	In 60 min	In 90 min	In 120 min
3.0	C	289	245, 151, 139, 123			X	X
1.5	GC	305	219, 203, 167, 125			X	X
5.8	Dimer (F-C)	561	561, 409, 289, 161	X	X	X	X
4.2	Dimer (F-GC/R-C)	577	577, 425, 409, 407, 305, 177, 161	X	X	X	X
2.6	Dimer (R-GC)	593	593, 425, 407, 305, 177			X	X
10.9	Trimer (F-C-F)	833	833, 681, 561, 289, 161	X	X	X	X
9.6	Trimer (F-C-R/F-GC-F)	849	849, 697, 561, 409, 305, 289, 177			X	X
7.9	Trimer (R-C-R)	865	865, 713, 577, 407, 289, 177		X	X	X
5.9	Trimer (R-GC-R)	881	880, 713, 593, 407, 305, 177		X	X	X

R, F, C, and GC represent robinetinidol, fisetinidol, catechin, and gallocatechin catechin, respectively. X signifies that the compound is regarded as present in the sample.

**Table 3:** Changes in antioxidant activity of *A. mearnsii* bark extract determined by DPPH and ABTS assays at different time points during in vitro gastrointestinal digestion.

Incubation Time (min)	DPPH IC_50_ (nL)			ABTS IC_50_ (nL)		
	Gastric Fraction	OUT Sample	IN Sample	Gastric Fraction	OUT Sample	IN Sample
0	6.9 ± 0.6a	9.7 ± 0.2a	0a	4.2 ± 0.3a	5.0 ± 0.4a	0a
30	7.2 ± 0.4b	16.4 ± 0.3b	120 ± 11a	4.4 ± 0.4b	14.8 ± 0.5a	71.1 ± 6a
60	7.8 ± 0.5a	15.7 ± 0.5c	85.6 ± 9a	4.7 ± 0.5c	11.7 ± 0.2a	36.9 ± 7a
90	7.4 ± 0.6c	11.5 ± 0.6d	57.3 ± 7a	4.5 ± 0.5d	9.9 ± 0.2a	18.8 ± 4a
120	7.3 ± 0.3d	10.1 ± 0.4e	47.4 ± 12a	4.5 ± 0.3e	5.9 ± 0.3a	11.4 ± 6a

The results were expressed as nanoliter of digested extract achieving half of the clearance rate. Values followed by the same letters (a–e) in the same column are significantly different from each other (*p* < 0.05). Data were means of three independent determinations ± SD.

## Supplementary Materials

The following materials are available online at http://www.mdpi.com/1420-3049/23/7/1804/s1. Table S1: Total polyphenol contents (TPC) of samples from *A. mearnsii* bark at different times during the simulated gastric-intestinal digestion without enzymes. Figure S1: Product ion scans (MS and MS^2^) of the *m*/*z* 289 molecular fragment in the IN sample. Figure S2: Product ion scans (MS and MS^2^) of the *m*/*z* 305 molecular fragment in the IN sample. Figure S3: Product ion scans (MS and MS^2^) of the *m*/*z* 561 molecular fragment in the IN sample. Figure S4: Product ion scans (MS and MS^2^) of the *m*/*z* 577 molecular fragment in the IN sample. Figure S5: Product ion scans (MS and MS^2^) of the *m*/*z* 593 molecular fragment in the IN sample. Figure S6: Product ion scans (MS and MS^2^) of the *m*/*z* 833 molecular fragment in the IN sample. Figure S7: Product ion scans (MS and MS^2^) of the *m*/*z* 849 molecular fragment in the IN sample. Figure S8: Product ion scans (MS and MS^2^) of the *m*/*z* 865 molecular fragment in the IN sample. Figure S9. Product ion scans (MS and MS^2^) of the *m*/*z* 881 molecular fragment in the IN sample.
