# Supplementary material for: Effects of In Vitro Digestion on the Content and Biological Activity of Polyphenols from Acacia mearnsii Bark
Source: Molecules. 2018 Jul 20;23(7):1804. doi: 10.3390/molecules23071804 (PMC6100292; doi:10.3390/molecules23071804)
Supplement: Supplementary file 1 [file molecules-23-01804-s001.pdf]

molecules

MDPI

Supplementary Material

Effects of In Vitro Digestion on the Content and Biological Activity of Polyphenols from *Acacia mearnsii* Bark

Xiao Chen [1], Jia Xiong [2], Lingxiao He [1], Yu Zhang [1], Xun Li [1], Liping Zhang [3] and Fei Wang [1,*]

[1] Jiangsu Key Laboratory for the Chemistry and Utilization of Agro-Forest Biomass, College of Chemical Engineering, Nanjing Forestry University, Nanjing 210037, China; 13770765711@163.com (X.C.); a6410@126.com (L.H.); yuzhang@njfu.edu.cn (Y.Z.); xunlee@163.com (X.L.)

[2] Plants for Human Health Institute, Food Bioprocessing and Nutrition Sciences Department, North Carolina State University, North Carolina Research Campus, NC 28081, USA; jxiong5@ncsu.edu

[3] College of Materials Sciences and Technology, Beijing Forestry University, Beijing 100083, China; zhanglp418@163.com

* Correspondence: hgwf@njfu.edu.cn; Tel.: +86-025-8542-7649

Abstract: The stability and bioaccessibility of polyphenol from *Acacia mearnsii* bark were measured at various stages during in vitro simulated digestion. Subsequently, the changes in the total polyphenol content (TPC) and biological activity were studied. The results showed that the phenolic compounds from *A. mearnsii* remained stable, and TPC underwent few changes during gastric digestion. Nonetheless, intestinal digestion led to the degradation of proanthocyanidins (PAs) and a significant decrease in TPC (26%). Degradation was determined by normal-phase HPLC and gel permeation chromatography. Only monomers, dimers, and trimers of flavan-3-ols were identified in the serum-accessible fraction for characterization of their bioaccessibility. The results also indicated the obvious antioxidant capacity of PAs from *A. mearnsii* bark, and ~53% of the α-glucosidase–inhibitory effect was preserved. All these findings show that PAs from *A. mearnsii* bark as a native plant source may be particularly beneficial for human health as a natural nutritional supplement.

Keywords: *Acacia mearnsii*; proanthocyanidins; in vitro digestion; antioxidant; antidiabetic; HPLC/MS

Contents

Table S1. Total polyphenol contents (TPC) of samples from *A. mearnsii* bark at different times during the simulated gastric-intestinal digestion without enzymes.

Figure S1. Product ion scan (MS and MS^2) of the m/z 289 in the IN sample.

Figure S2. Product ion scan (MS and MS^2) of the m/z 305 in the IN sample.

Figure S3. Product ion scan (MS and MS^2) of the m/z 561 in the IN sample.

Figure S4. Product ion scan (MS and MS^2) of the m/z 577 in the IN sample.

Figure S5. Product ion scan (MS and MS^2) of the m/z 593 in the IN sample.

Figure S6. Product ion scan (MS and MS^2) of the m/z 833 in the IN sample.

Figure S7. Product ion scan (MS and MS^2) of the m/z 849 in the IN sample.

Figure S8. Product ion scan (MS and MS^2) of the m/z 865 in the IN sample.

Figure S9. Product ion scan (MS and MS^2) of the m/z 881 in the IN sample.

Table S1. Total polyphenol contents (TPC) of samples from *A. mearnsii* bark at different times during the simulated gastric-intestinal digestion without enzymes.

Digestion Time (min)	TPC of Digestion Samples (mg/mL)		
	Gastric Digestion	OUT Sample	IN Sample
0	5.97 ± 0.05	5.09 ± 0.02	0
30	5.83 ± 0.06	5.06 ± 0.03	0.25 ± 0.02
60	5.88 ± 0.04	5.01 ± 0.04	0.68 ± 0.02
90	5.91 ± 0.03	4.97 ± 0.02	1.23 ± 0.03
120	5.89 ± 0.04	4.99 ± 0.04	1.74 ± 0.04

The IN sample represents the solution that diffused into the dialysis tubing, the OUT sample represents the solution outside of the tubing. Data represent the means of three independent determinations ± SD.

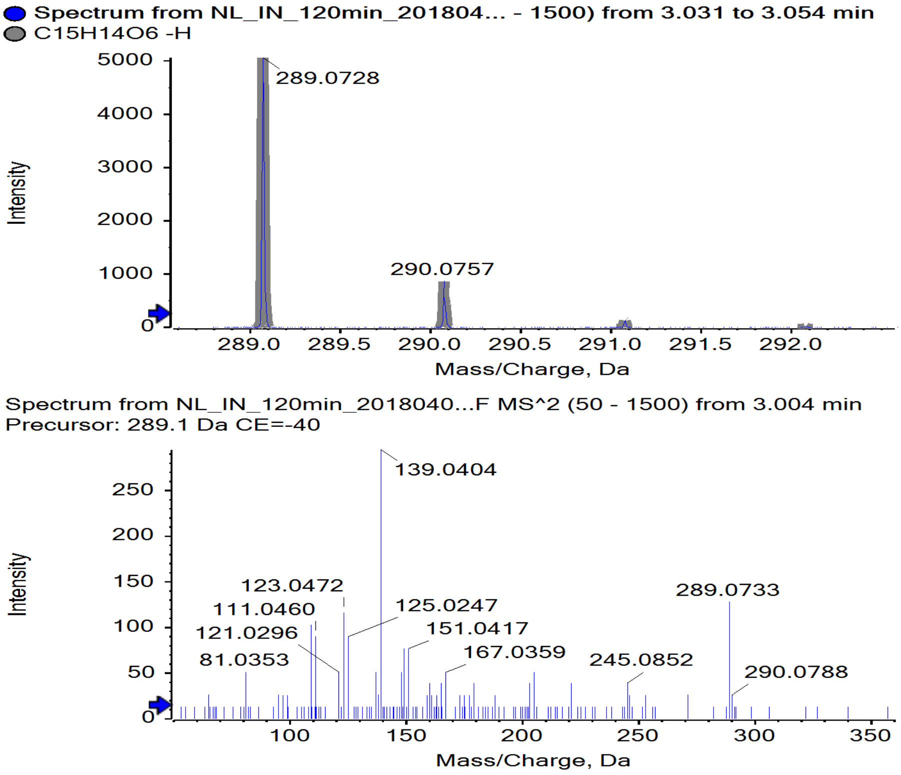

Figure S1. Product ion scan (MS and MS^2) of the m/z 289 in the IN sample.

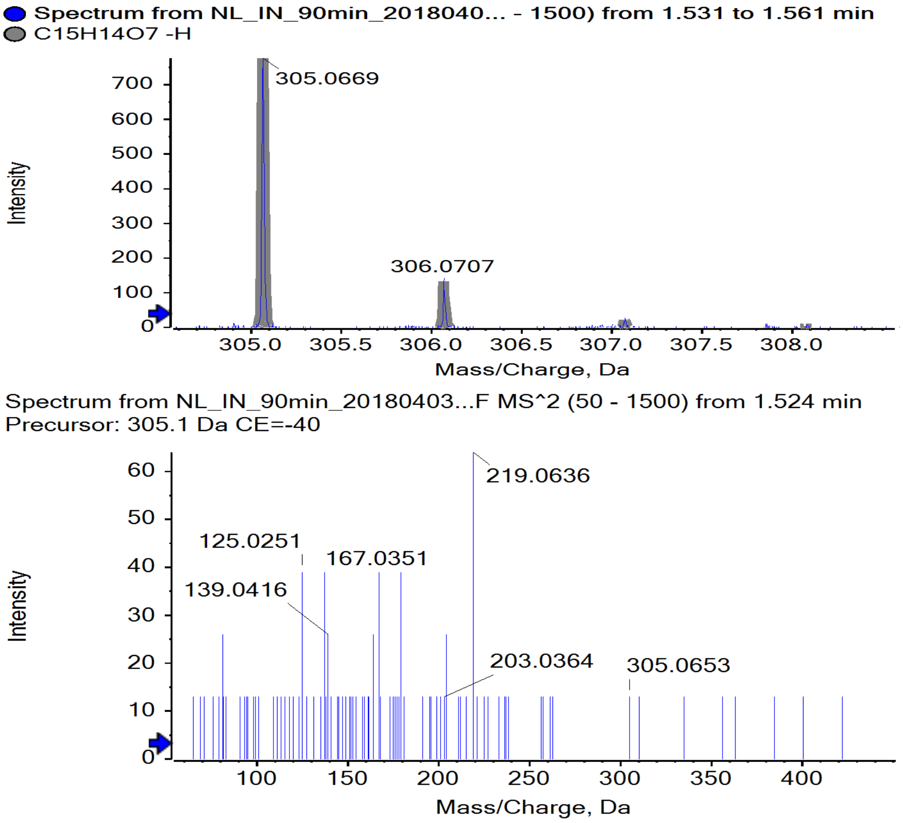

Figure S2. Product ion scan (MS and MS²) of the m/z 305 fragment in the IN sample.

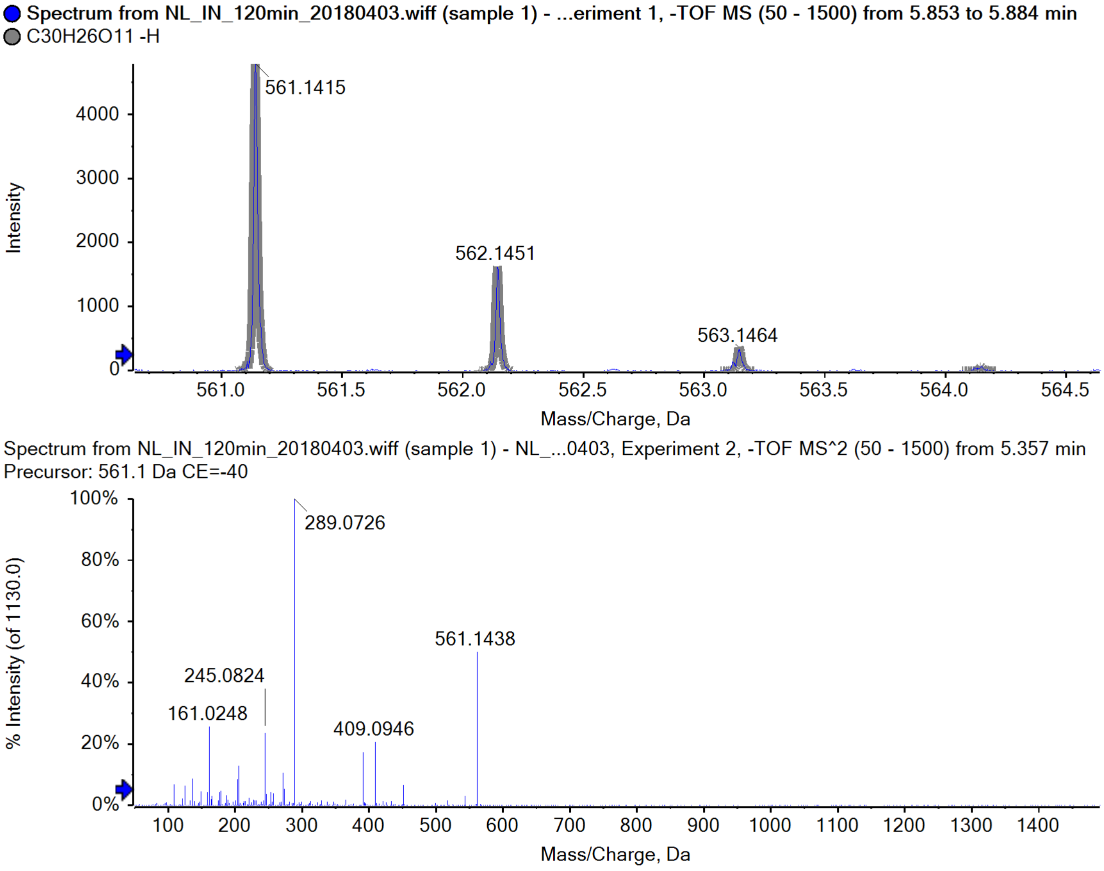

Figure S3. Product ion scan (MS and MS²) of the m/z 561 in the IN sample.

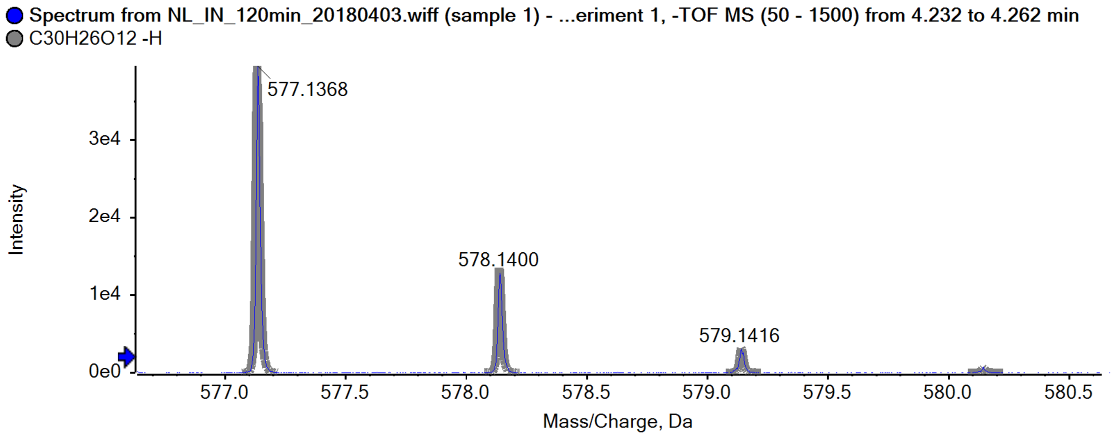

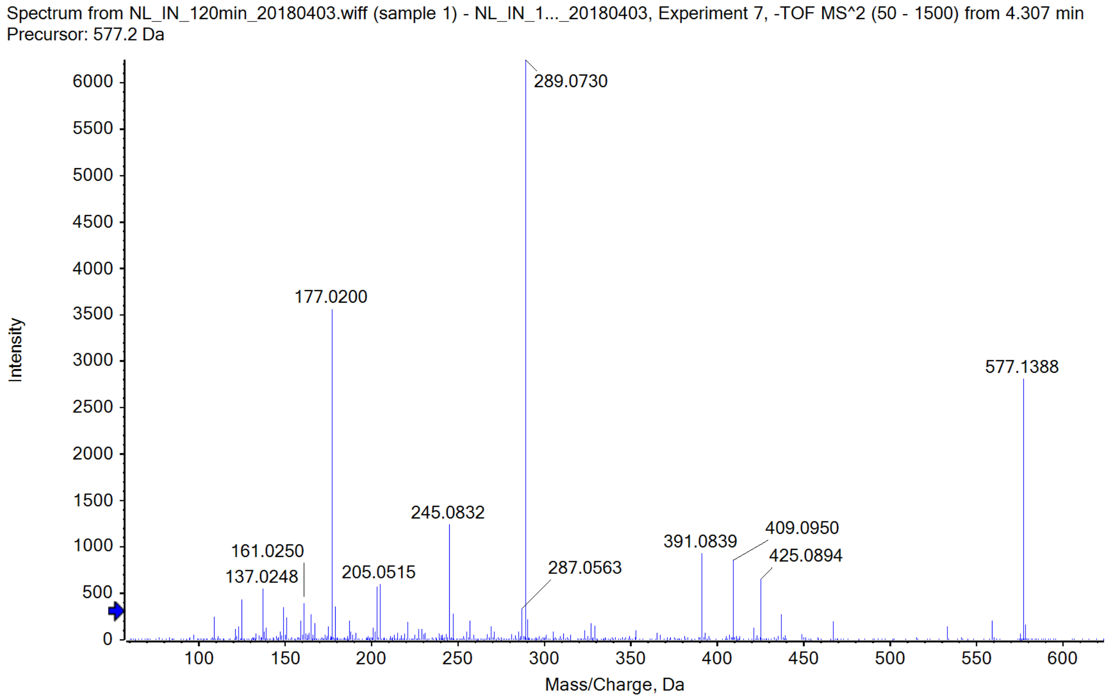

Figure S4. Product ion scan (MS and MS²) of the m/z 577 in the IN sample.

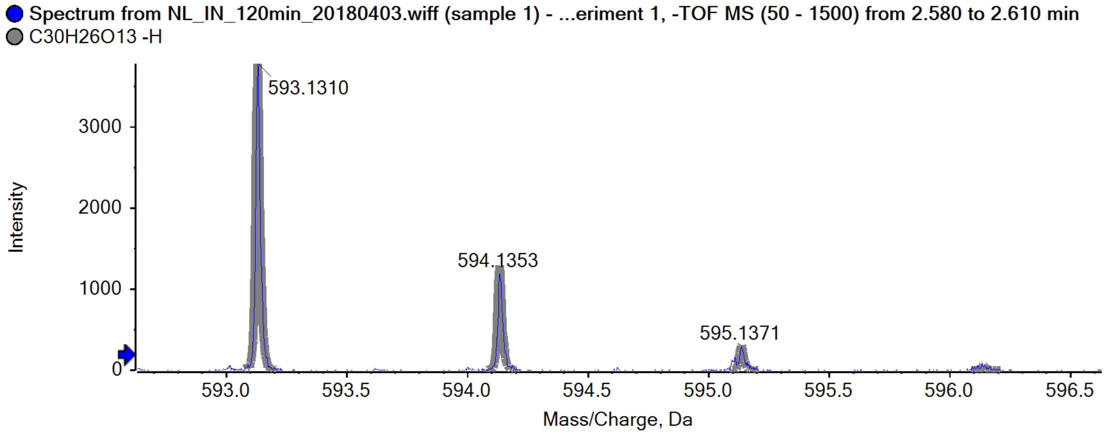

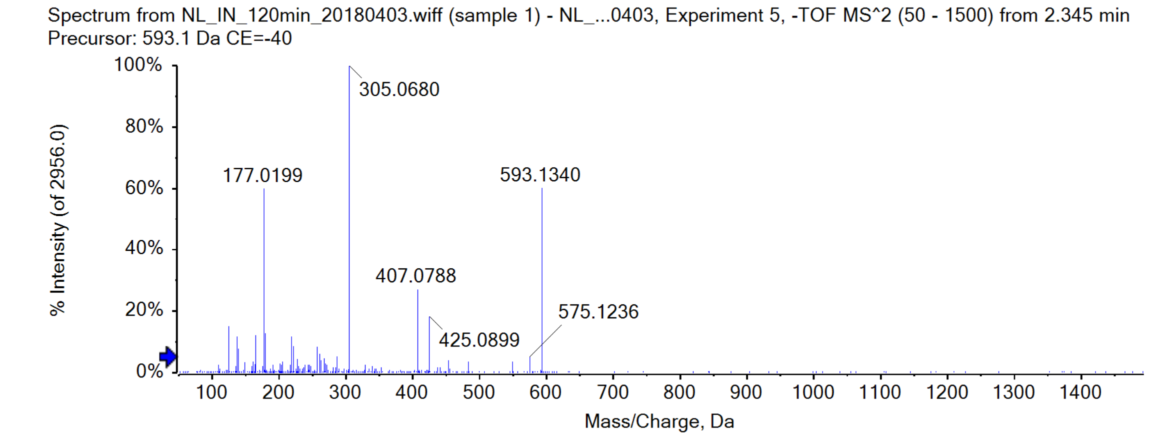

Figure S5. Product ion scan (MS and MS²) of the m/z 593 in the IN sample.

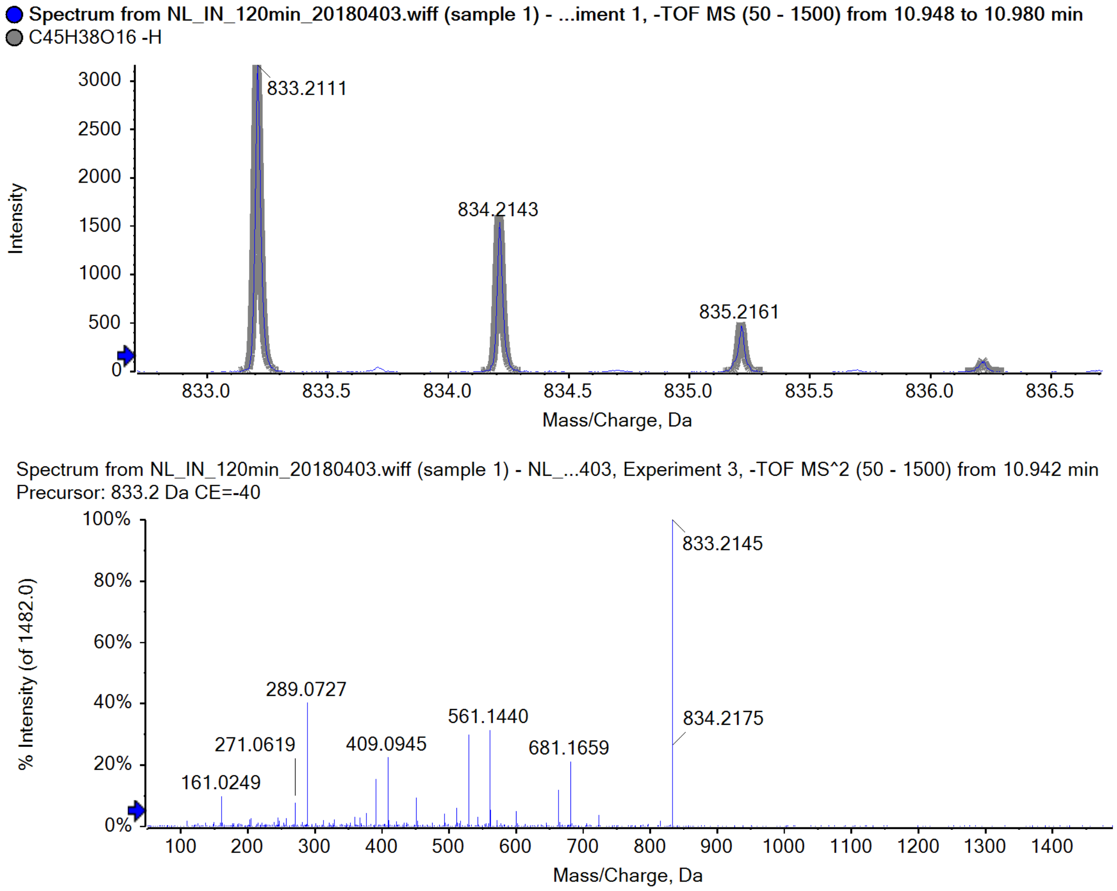

Figure S6. Product ion scan (MS and MS²) of the m/z 833 in the IN sample.

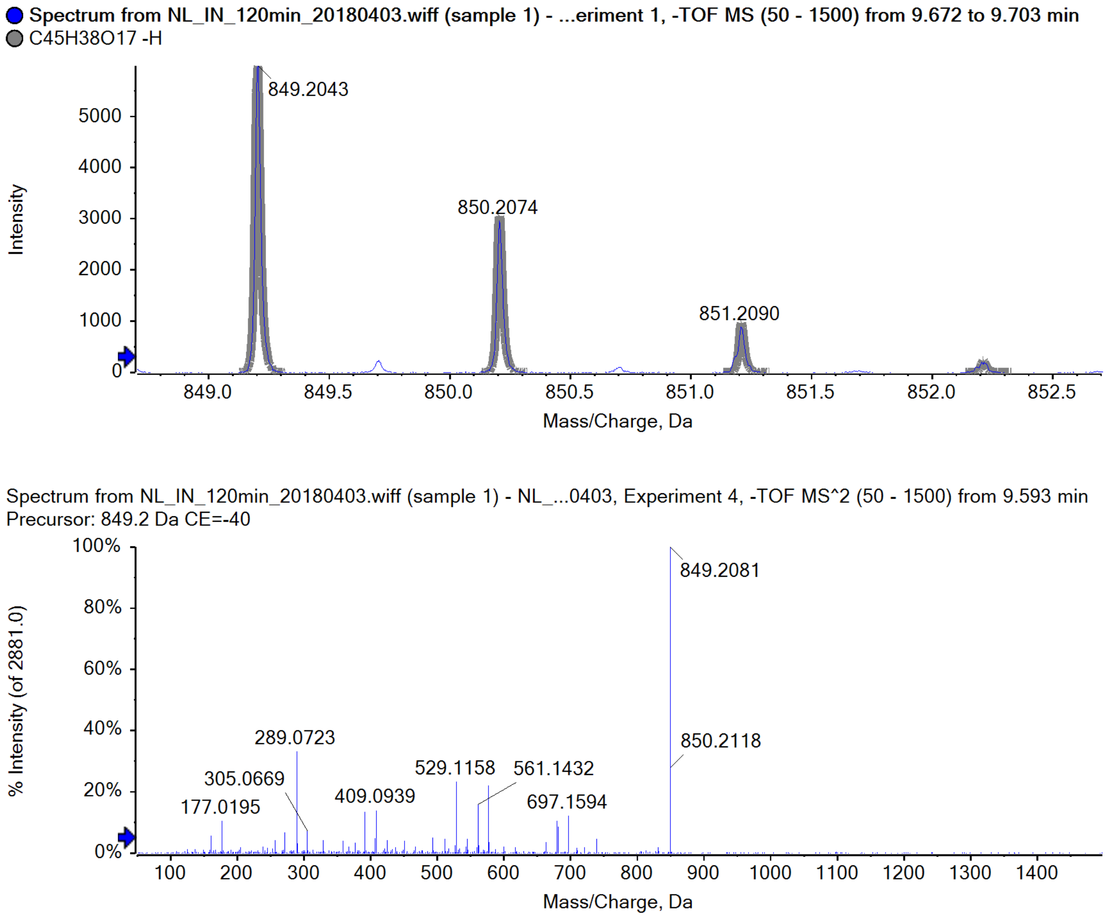

Figure S7. Product ion scan (MS and MS²) of the m/z 849 in the IN sample.

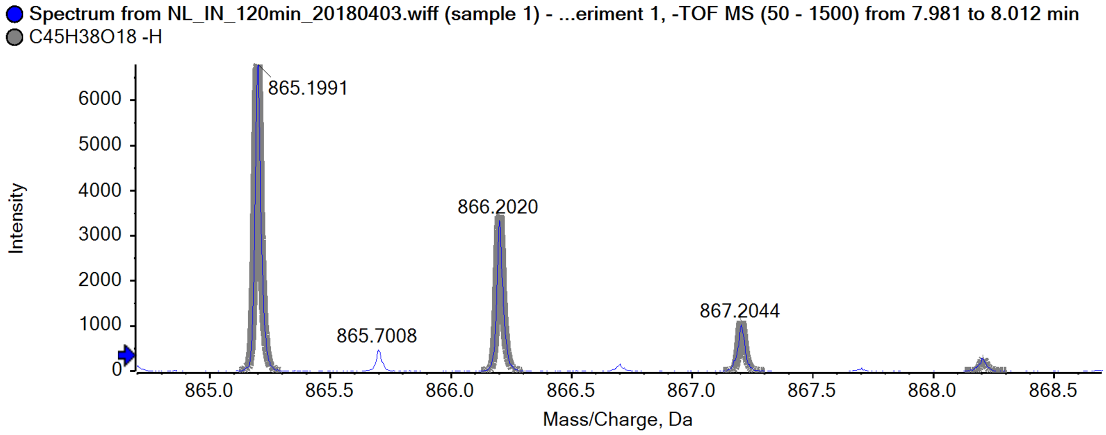

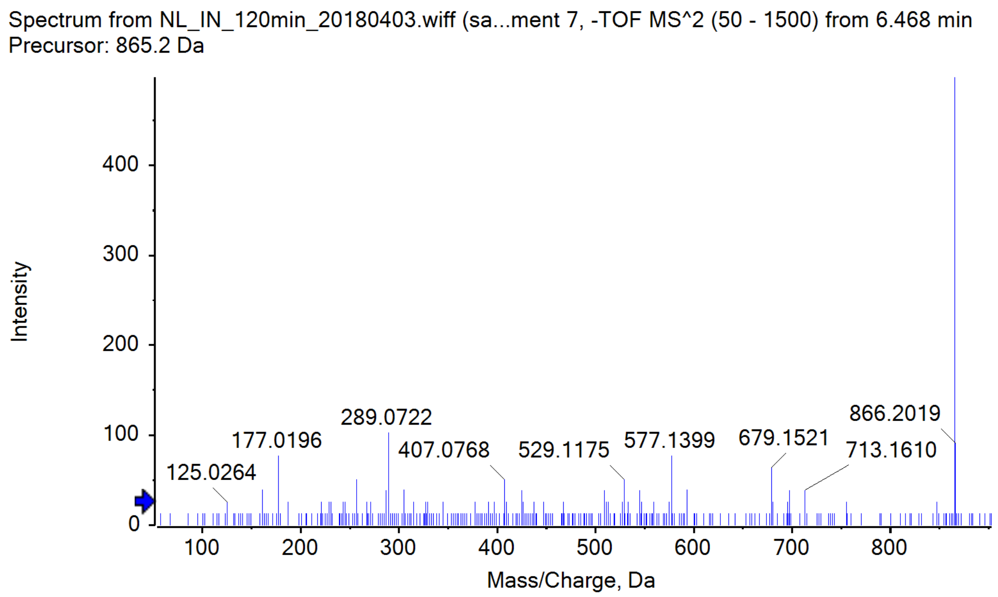

Figure S8. Product ion scan (MS and MS[2]) of the m/z 865 in the IN sample.

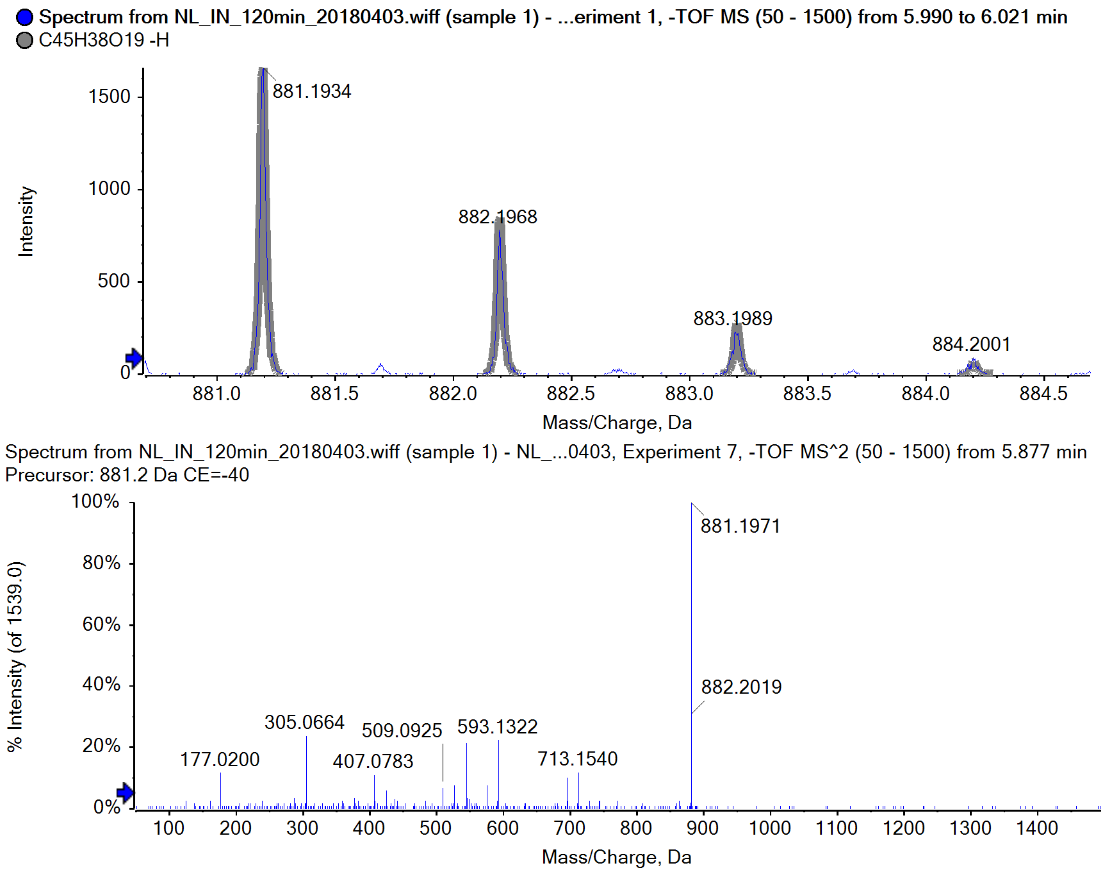

Figure S9. Product ion scan (MS and MS[2]) of the m/z 881 in the IN sample.
